# Ureolytic Prokaryotes in Soil: Community Abundance and Diversity

**DOI:** 10.1264/jsme2.ME17188

**Published:** 2018-04-28

**Authors:** Mamoru Oshiki, Mitsuru Araki, Yuga Hirakata, Masashi Hatamoto, Takashi Yamaguchi, Nobuo Araki

**Affiliations:** 1 Department of Civil Engineering, National Institute of Technology, Nagaoka College Nagaoka Japan; 2 Department of Science of Technology Innovation, Nagaoka University of Technology Nagaoka Japan; 3 Department of Environmental Systems Engineering, Nagaoka University of Technology Nagaoka Japan

**Keywords:** urea turnover, urease, *ur*e*C*, ureolytic prokaryote, soil microbiology

## Abstract

Although the turnover of urea is a crucial process in nitrogen transformation in soil, limited information is currently available on the abundance and diversity of ureolytic prokaryotes. The abundance and diversity of the soil 16S rRNA gene and *ur*e*C* (encoding a urease catalytic subunit) were examined in seven soil types using quantitative PCR and amplicon sequencing with Illumina MiSeq. The amplicon sequencing of *ur*e*C* revealed that the ureolytic community was composed of phylogenetically varied prokaryotes, and we detected 363 to 1,685 species-level *ur*e*C* operational taxonomic units (OTUs) per soil sample, whereas 5,984 OTUs were site-specific OTUs found in only one of the seven soil types.

Urea is a nitrogenous component of soil and is supplied via various emission routes: *i.e.*, mammalian urine; uric acid from birds, amphibians, and insects (10), and the application of a urea-based chemical fertilizer (12). Regarding urea-based fertilizers, 60% of the annual consumption of nitrogenous fertilizers is currently derived from urea consumption (7). The turnover of urea plays a crucial role in nitrogen transformation in the rhizosphere and agricultural soil because urea is produced during the mineralization of organic nitrogenous compounds (*i.e.*, the degradation of amino acids and nucleotides), and its turnover accounts for 60–200% of gross nitrogen mineralization (16). The rate of urea hydrolysis without biological activity is only 0–2% of biological activity in ambient temperatures (23); therefore, microorganisms are strongly involved in urea hydrolysis. Urea hydrolysis is performed by prokaryotic urease (Ure) and ATP:urea amidolyase (EC 6.3.4.6) of yeast and algae (14), and urea hydrolysis by prokaryotic urease is considered to be the predominant pathway (23). Soil matrix-bound (*i.e.*, extracellular) and intracellular ureases are both involved in urea hydrolysis in soil (16, 21), and prokaryotic urease is composed of two or three catalytic subunits (UreA, UreB, and UreC) (20). *ur*e*C* has been used as a functional gene marker for the detection of ureolytic prokaryotes in grassland soil (21), oceans (4), marine sponges (22), rumen (13), ureolytic bioreactors (9), and ground water (8) because it contains several conserved regions. The ureolytic prokaryotic community in grassland soil was previously examined in a PCR-cloning-sequencing analysis, and *Bradyrhizobium*-, *Bacillus*-, *Methylobacter* spp.-, and *Flavobacterium*-related *ur*e*C* sequences were retrieved (21). These findings provided an insight into the ureolytic-prokaryotic-community composition of grassland soil, whereas it was not possible to investigate the ureolytic prokaryotic community in detail using a rarefaction analysis. It is also important to note that previous studies (9, 13, 21) used the oligonucleotide primers ureC_F and ureC_R (19) for the PCR amplification of *ur*e*C*, whereas our sequence alignment revealed that these primers contained several primer–template mismatches (see below). Moreover, there is currently no information on the ureolytic-prokaryotic-community structure other than that in grassland soil; therefore, the community structure of ureolytic prokaryotes in soil remains largely unknown. In the present study, we aimed to investigate the abundance and diversity of *ur*e*C* sequences in various types of soils using a set of proper *ur*e*C* primers and amplicon sequencing on an Illumina MiSeq sequencer.

Soil samples were collected at seven sites in Nagaoka city, Japan (Table S1), including agricultural (Soil 1), forest (Soils 2 and 7), grassland (Soil 3), urban park (Soil 4), and compost (Soil 5) soil as well as freshwater sediment (Soil 6). These sampling sites were selected in order to examine the abundance and diversity of ureolytic prokaryotes in various soil types with different physicochemical characteristics (Table S1). Surface layers (0 to 5 cm) were collected from five spots at each site (5×1 m), mixed, and sieved (pore diameter of 5 mm) to remove concomitant gravel. Sieved soil samples were subjected to the following batch incubation and DNA extraction. The ureolytic rate was assessed by incubating soil samples aerobically in triplicate as previously described by Fisher *et al.* (5). Briefly, 2 g of wet soil was resuspended in 10 mL of an inorganic medium containing 794 μM N urea, and incubated at 15°C with shaking at 100 rpm. The pH of the soil suspension depended on the pH of the soil samples collected, which ranged between pH 5.1 and 9.4 as shown in Table S1. Urea and NH_3_ concentrations in soil suspensions were assessed during the incubation by a colorimetric method using diacetyl monoxime (2) and phenol (17), respectively. All the soil samples studied hydrolyzed urea to ammonia (Fig. S1) at a rate of 0.075 to 0.123 μmol g dry soil^−1^ h^−1^ (Fig. 1), indicating that ureolytic prokaryotes were widespread in the soil samples studied. Notably, the urea concentration in the batch incubation (*i.e.*, 794 μM N) was similar to that found in soil supplemented with a urea-based fertilizer (*i.e.* 100 mg N kg soil^−1^), but was an order of magnitude lower than the *K_m_* value of bacterial urease (typically in the millimolar range) (9). Therefore, the above ureolytic rates may underestimate the potential ureolytic rates of soil.

The abundance of *ur*e*C* and the 16S rRNA gene in the soil samples studied was assessed using a quantitative PCR (qPCR) assay. The oligonucleotide primers ureC_F and ureC_R (19) have generally been used in the PCR amplification of *ur*e*C* (9, 13, 21); however, these primers contained several primer–template mismatches with the known *ur*e*C* sequences (Fig. S2a and b). Therefore, we selected the L2F_V1 (5′-CGGCA AGGCCGGCAACCC-3′) and 733R (5′-GTBGHDCCCCAR TCYTCRT-3′) primers (6) (amplicon size; *ca.* 386 bp) for the PCR amplification of *ur*e*C*, and these primers showed higher sequence coverage than ureC_F and ureC_R (Fig. S2c and d). The sequence of the primer L2F_V1 was taken from the primer L2F (6) with the following minor modifications to decrease sequence complexity, *i.e.*, H to C at position +3, Y to C at +6, R to G at +9, N to C at +12, N to C at +15, and Y to C at +18. This decrease in sequence complexity enabled the specific amplification of *ur*e*C* in the soil samples studied. Genomic DNA was extracted from sieved soil samples, and qPCR assays using the 515F and 806R primers for the 16S rRNA gene (1) and the L2F_V1 and 733R primers for *ureC* were performed (See Supplementary text for the detailed protocol.). qPCR assays were performed in triplicate, and the abundance of the 16S rRNA gene and *ureC* were 5.5×10^7^ to 2.1×10^9^ copies g dry soil^−1^ and 9.5×10^6^ to 3.8×10^7^ copies g dry soil^−1^, respectively (Fig. 1). The relative abundance of the copy numbers of *ur*e*C* toward those of the 16S rRNA gene differed among the seven soil samples, and ranged between 1.4% and 17%. Pearson’s correlation analysis revealed that the 16S rRNA gene and *ureC* abundance both correlated (*P*<0.05) with ureolytic rates (*R^2^*=0.719 and 0.578, respectively). The weak correlation between *ur*e*C* abundance and ureolytic rates may have been due to the overestimation of metabolically-active ureolytic prokaryotes in the soil samples examined, and may have been caused by DNA extraction from dead and dormant cells in soil.

The prokaryotic community structure and ureolytic community structure of the soil samples examined were analyzed by the amplicon sequencing of the 16S rRNA gene and *ur*e*C* using Illumina MiSeq. PCR amplification of the 16S rRNA gene and *ur*e*C* was performed using the above-described oligonucleotide primers containing Illumina tag sequences at the 5′ end of the forward and reverse primers (5′-TCGTCGG CAGCGTCAGATGTGTATAAGAGACAG-3′ and 5′-GTC TCGTGGGCTCGGAGATGTGTATAAGAGACAG-3′, respectively). PCR products were tagged with a sampleunique index and Illumina adapter sequences at their 5′ end (Nextera XT Index Kit v2; Illumina, San Diego, CA, USA) by PCR, and sequenced on the Illumina MiSeq platform in a 250-bp paired-end sequencing reaction with the v2 reagent kit (Illumina). The *ur*e*C* and 16S rRNA gene sequence reads generated were processed for the removal of adapter sequences using cutadapt and for quality trimming using Trimmomatic v0.33, as previously described (11) (Supplementary text). The 6,638 to 15,342 sequence reads of the 16S rRNA gene and 2,511 to 6,248 sequence reads of *ur*e*C* were obtained from each soil sample. Regarding the 16S rRNA gene, sequence reads were clustered based on ≥97% sequence identity into 1,143 to 2,263 operational taxonomic units (OTUs) (Table S2a), and bacterial members affiliated with the phyla *Proteobacteria*, *Acidobacteria*, *Verrucomicrobia*, *Bacteroidetes*, and *Cyanobacteria* were distributed in all soil samples examined (Fig. S3). Regarding the sequence reads of *ur*e*C*, we needed to set a threshold of sequence identity below which *ur*e*C* reads were assumed to come from different bacterial species prior to clustering of the sequence reads into OTUs. This action was necessary in order to examine the diversity of ureolytic prokaryotes. Therefore, the sequence identities of *ur*e*C* and the 16S rRNA gene located in 69 alphaproteobacterial genomes were examined using blastn searches with *ur*e*C* (corresponding to nucleotide positions 294 to 680, the region amplified using the primers L2F_V1 and 733R) and 16S rRNA gene sequences (nucleotide positions 10 to 1,464, the conserved region amplified with the primers 27F and 1492R [15]) in the *Bradyrhizobium lablabi* genome (accession number LT670845) as a query sequence. The 99% sequence identity of the 16S rRNA gene has conventionally served as a threshold for distinguishing bacterial species (3), and the 93.3% identity of *ur*e*C* corresponded to the 99% identity of the 16S rRNA gene, as shown in Fig. S4. Apart from the alphaproteobacterial *ur*e*C*, correlations between gamma- and beta-proteobacterial 16S rRNA genes and gamma- and beta-proteobacterial *ur*e*C* were also examined using gamma-(*i.e.*, *Cobetia marina* genome, NZ_CP017114.1) and betaproteobacterial (*Cupriavidus gilardii* genome, NZ_CP010516) genomes as query sequences. The 93.6% and 89.0% identities of *ur*e*C* corresponded to the 99% identity of the 16S rRNA gene. The average identity of *ur*e*C* corresponding to the 99% identity of the 16S rRNA gene was 92.0%; therefore, we selected 91% nucleic acid sequence identity as a conservative threshold value (18), and used it for clustering our *ur*e*C* sequence reads into species-level OTUs. The retrieved sequence reads of *ur*e*C* were grouped into 363 to 1,685 species-level OTUs per soil sample (Table S2b) and 6,852 OTUs in total. Although we obtained more than 2,511 *ur*e*C* reads per soil sample, Good’s coverage was often less than 90% (Table S2), indicating the high diversity of the soil ureolytic prokaryotic community. Difficulties have been associated with examining the diversity of the soil ureolytic prokaryotic community structure using previous PCR-DGGE-(9) and PCR-cloning-sequencing (4, 21, 22) analyses in which fewer than 96 *ur*e*C* sequences were examined per sample. To the best of our knowledge, this is the first study to describe a soil ureolytic prokaryotic community in detail. Notably, the ureolytic-community structure in soil was mainly composed of site-specific OTUs. Only 6 out of 6,852 species-level OTUs of *ur*e*C* and 123 species-level OTUs of the 16S rRNA gene were shared among the soil samples examined.

On the other hand, the community structure of ureolytic prokaryotes was investigated in the present study using amplicon sequencing without biological replicates; therefore, further verification studies are required in order to clarify how widespread site-specific OTUs are.

The phylogenies of the dominant species-level OTUs of *ur*e*C* were examined in order to identify dominant ureolytic prokaryotes in soil. The 6,852 species-level OTUs of *ur*e*C* detected were arranged in order of the abundance of *ur*e*C* reads obtained from the seven soil samples, and, thus, the 34 most abundant species-level OTUs of *ur*e*C* were selected. A phylogenetic tree was constructed using the representative nucleic acid sequences of the 34 most abundant species-level OTUs of *ur*e*C*, and these *ur*e*C* OTUs were found to be affiliated with phylogenetically diverse *ur*e*C* clades, including those of *α-*, *β-*, and *γ-Proteobacteria*; *Firmicutes*; *Actinobacteria*; *Nitrospira*; *Chloroflexi*; *Cyanobacteria*; and *Archaea* (Fig. 2). Sequence identities between the *ur*e*C* sequences of the 34 most abundant species-level OTUs of *ur*e*C* and known *ur*e*C* sequences are shown in Table S3. Twenty-nine out of the 34 most abundant species-level OTUs of *ur*e*C* showed a sequence identity lower than 91%, and the *ur*e*C* sequence of the closest relative was derived from the prokaryotic genome and not from environmental clones other than *ur*e*C* OTU3157, OTU4155, and OTU4606 (Table S3). These results indicate that current knowledge on environmental *ur*e*C* sequences is limited, even in relation to dominant ureolytic prokaryotes in soil. Notably, the phylogeny of *ur*e*C* was occasionally incongruent with that of the 16S rRNA gene: *e.g.*, a clade of *Burkholderia*-related *ur*e*C* was separated from that of other *β-proteobacterial ur*e*C* (Fig. 2). Bacterial *ur*e*C* may be transferred between bacterial genomes (4, 22); this phenomenon may have contributed to incongruence.

In summary, ureolytic prokaryotes are distributed in various types of soils, and this study is the first to describe the abundance and diversity of these functional microorganisms in several soil types. The soil ureolytic prokaryotic community is composed of phylogenetically diverse members, and each soil type has a unique ureolytic-prokaryotic-community composition. Additional studies are needed in order to investigate the distribution of ureolytic prokaryotes and identify the physicochemical parameters regulating the abundance and diversity of these prokaryotes.

## Supplementary Material



## Figures and Tables

**Fig. 1:**
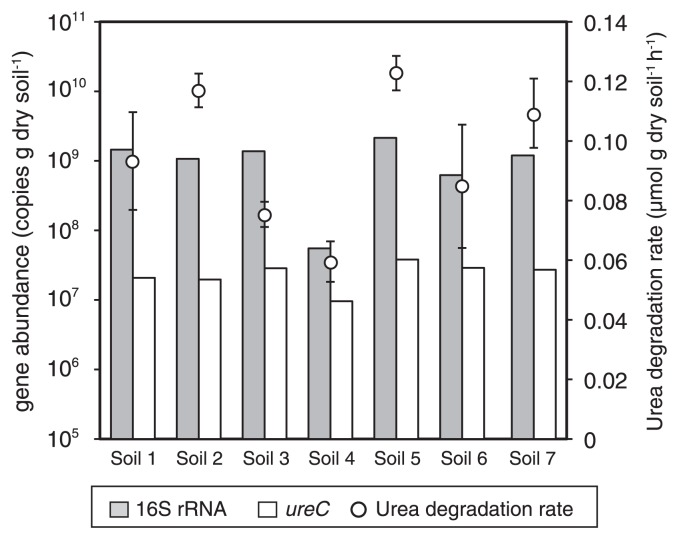
Urea degradation rates (circles) and abundance of the *ur*e*C* gene (white bars) and 16S rRNA gene (grey bars). The abundance of the genes was shown as mean values of triplicate qPCR assays.

**Fig. 2:**
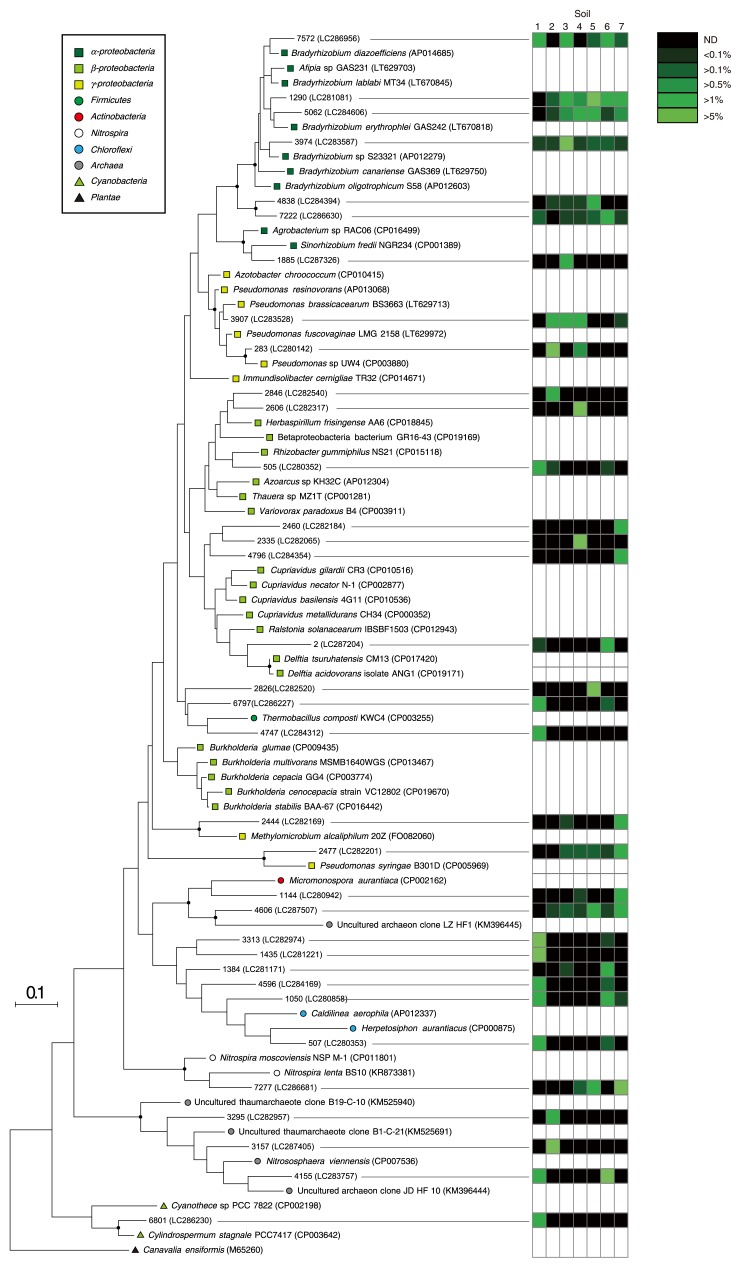
Phylogeny of 34 most abundant operational taxonomic units (OTUs) of *ur*e*C* (corresponding to the species level). *ur*e*C* reads obtained by amplicon sequencing on the Illumina MiSeq platform were clustered into species-level OTUs with ≥91% sequence identity, and a phylogenetic tree was constructed by the maximum likelihood method with the Jones-Taylor-Thornton model by means of the *ur*e*C* sequence of *Canavalia ensiformis* (M65260) as an outgroup. Branching points that support probability >80% in the bootstrap analyses (based on 500 replicates) are shown as filled circles. The heatmap shows the relative abundance of species-level OTUs of *ure*C in soil samples 1 to 7. The scale bar represents 20% sequence divergence. Nucleotide sequence accession numbers are indicated in parentheses.
