# Supplementary material for: Ureolytic Prokaryotes in Soil: Community Abundance and Diversity
Source: Microbes Environ. 2018 Apr 28;33(2):230–3. doi: 10.1264/jsme2.ME17188 (PMC6031400; doi:10.1264/jsme2.ME17188)
Supplement: Supplementary file 1 [file 33_230_s1.pdf]

Supplementary material

This manuscript contains 4 supplementary figure, 3 supplementary table and 1 supplementary text, respectively. Those can be found in the online version of this article on the publisher's website.

Supplementary Figure Legends

Figure S1. Urea hydrolysis by the studied soil sample. Soil 2 was aerobically incubated with urea (794 µM-N), and urea (circles) and ammonia concentrations (squares) were determined during 36 h of incubation. Error bars indicate the range of standard deviations derived from triplicate incubations.

Figure S2. Primer–temperate mismatches between the previously developed oligonucleotide *ur*e*C* primers and prokaryotic *ur*e*C* sequences. Alignment positions of primers ureC_F, ureC_R, L2F_V1, and 733R (panels a, b, c, and d, respectively) in the *Bradyrhizobium lablabi ur*e*C* gene (LT670845) were 646, 970, 294, and 680, respectively. The 2,653 *ur*e*C* sequences located in prokaryotic genomes were retrieved from the IMC genome database, aligned, and the mismatches were counted. The row "Coverage" indicates sequence coverage of the examined *ur*e*C* primer at the specific position, and the heatmap highlights the regions showing high sequence coverage, i.e. red for >90% coverage, pink for >75% coverage, and yellow for >50% coverage.

Figure S3. Taxonomic classification of prokaryotic communities in soils. The heatmap indicates the relative abundance of 16S rRNA gene reads. The reads that are not classified into any known prokaryotic group are labelled as 'other'. The complete dendrogram of linkage hierarchical clusters among the prokaryotic community structures in the seven soils is shown in the upper panel.

Figure S4. Correlation between the identity of α-proteobacterial *ureC* and 16S rRNA gene sequences.** The *ur*e*C* identity and 16S rRNA identity were assessed by means of the blastn program using *ur*e*C* (corresponding to nucleotide positions 294 to 680) and 16S rRNA gene sequence (nucleotide positions 10 to 1,464) in the *Bradyrhizobium lablabi* genome (accession number LT670845) as query sequences and the *ur*e*C* and 16S rRNA gene sequences located in the α-proteobacterial genome deposited in the National Center of Biotechnology Information (NCBI) database as reference sequences, respectively. Note that 93.3% identity of *ur*e*C* corresponds to 99% identity of the 16S rRNA gene as determined by linear regression analysis; 91% identity was used in the present study as a conservative threshold value for grouping the *ur*e*C* sequence reads.

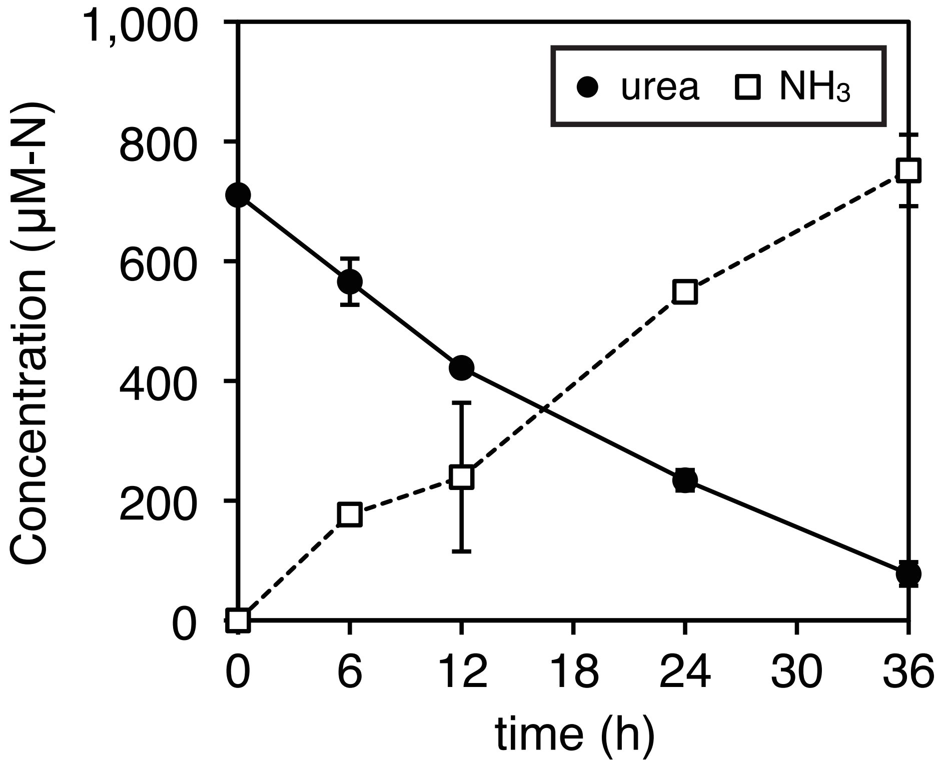
1,000
800
600
400
200
0
Concentration (μM-N)
0
6
12
18
24
30
36
time (h)
urea
NH3

Figure S1

a) ureC_F

		1	2	3	4	5	6	7	8	9	10	11	12	13	14	15	16	17	18	19	20	21	22	23	24	25	26	27
primer (5' - 3')		T	G	G	G	C	C	T	T	A	A	A	A	T	H	C	A	Y	G	A	R	G	A	Y	T	G	G	G
ureC alignment	A	0%	5%	1%	0%	9%	2%	2%	10%	100%	100%	28%	14%	0%	4%	0%	100%	0%	0%	100%	42%	0%	100%	0%	0%	0%	0%	0%
	T	60%	15%	8%	0%	16%	32%	98%	5%	0%	0%	0%	7%	100%	10%	0%	0%	31%	0%	0%	0%	0%	0%	20%	99%	1%	1%	0%
	G	25%	17%	91%	81%	32%	1%	0%	49%	0%	0%	72%	9%	0%	62%	0%	0%	0%	100%	0%	58%	100%	0%	0%	1%	99%	99%	100%
	C	15%	63%	0%	19%	43%	65%	0%	36%	0%	0%	0%	70%	0%	23%	100%	0%	69%	0%	0%	0%	0%	0%	80%	0%	0%	0%	0%
Coverage		60%	17%	91%	81%	43%	65%	98%	5%	100%	100%	28%	14%	100%	38%	100%	100%	100%	100%	100%	100%	100%	100%	100%	99%	99%	99%	100%

b) ureC_R

		1	2	3	4	5	6	7	8	9	10	11	12	13	14	15	16	17	18	19	20	21	22	23	24	25
primer (5' - 3')		G	G	T	G	G	T	G	G	C	A	C	A	C	C	A	T	N	A	N	C	A	T	R	T	C
ureC alignment	A	12%	25%	0%	0%	43%	0%	0%	22%	1%	97%	12%	98%	1%	0%	100%	0%	3%	98%	15%	0%	100%	0%	22%	0%	0%
	T	0%	0%	99%	3%	0%	100%	0%	1%	0%	1%	6%	0%	2%	0%	0%	98%	4%	0%	7%	0%	0%	100%	0%	99%	0%
	G	87%	74%	0%	95%	57%	0%	100%	77%	1%	0%	47%	1%	0%	2%	0%	0%	23%	2%	72%	0%	0%	0%	78%	0%	0%
	C	0%	1%	0%	1%	0%	0%	0%	0%	97%	1%	35%	0%	97%	98%	0%	2%	70%	0%	5%	100%	0%	0%	1%	1%	99%
Coverage		87%	74%	99%	95%	57%	100%	100%	77%	97%	97%	35%	98%	97%	98%	100%	98%	100%	98%	100%	100%	100%	100%	99%	99%	99%

c) L2F_V1

		1	2	3	4	5	6	7	8	9	10	11	12	13	14	15	16	17	18
primer (5' - 3')		C	G	G	C	A	A	G	G	C	C	G	G	C	A	A	C	C	C
ureC alignment	A	6%	0%	0%	5%	96%	96%	28%	3%	0%	9%	0%	0%	8%	100%	100%	0%	0%	0%
	T	14%	0%	0%	13%	0%	0%	1%	2%	0%	8%	0%	0%	14%	0%	0%	38%	1%	0%
	G	5%	100%	100%	5%	0%	3%	69%	95%	4%	24%	100%	100%	6%	0%	0%	0%	0%	0%
	C	75%	0%	0%	77%	4%	0%	2%	0%	96%	59%	0%	0%	71%	0%	0%	62%	99%	100%
Coverage		75%	100%	100%	77%	96%	96%	69%	95%	96%	59%	100%	100%	71%	100%	100%	62%	99%	100%

d) 733R

		1	2	3	4	5	6	7	8	9	10	11	12	13	14	15	16	17	18	19
primer (5' - 3')		G	T	B	G	H	D	C	C	C	C	A	R	T	C	Y	T	C	R	T
ureC alignment	A	2%	1%	6%	0%	17%	11%	0%	0%	1%	1%	99%	20%	0%	0%	0%	0%	0%	31%	0%
	T	0%	98%	11%	0%	64%	12%	0%	0%	0%	0%	0%	0%	100%	0%	42%	100%	0%	0%	100%
	G	98%	0%	47%	96%	0%	69%	0%	0%	0%	0%	0%	80%	0%	0%	0%	0%	0%	69%	0%
	C	0%	0%	35%	4%	19%	9%	100%	100%	99%	98%	1%	0%	0%	100%	58%	0%	100%	0%	0%
Coverage		98%	98%	94%	96%	100%	91%	100%	100%	99%	98%	99%	100%	100%	100%	100%	100%	100%	100%	100%

>90% >75% >50%

Figure S2

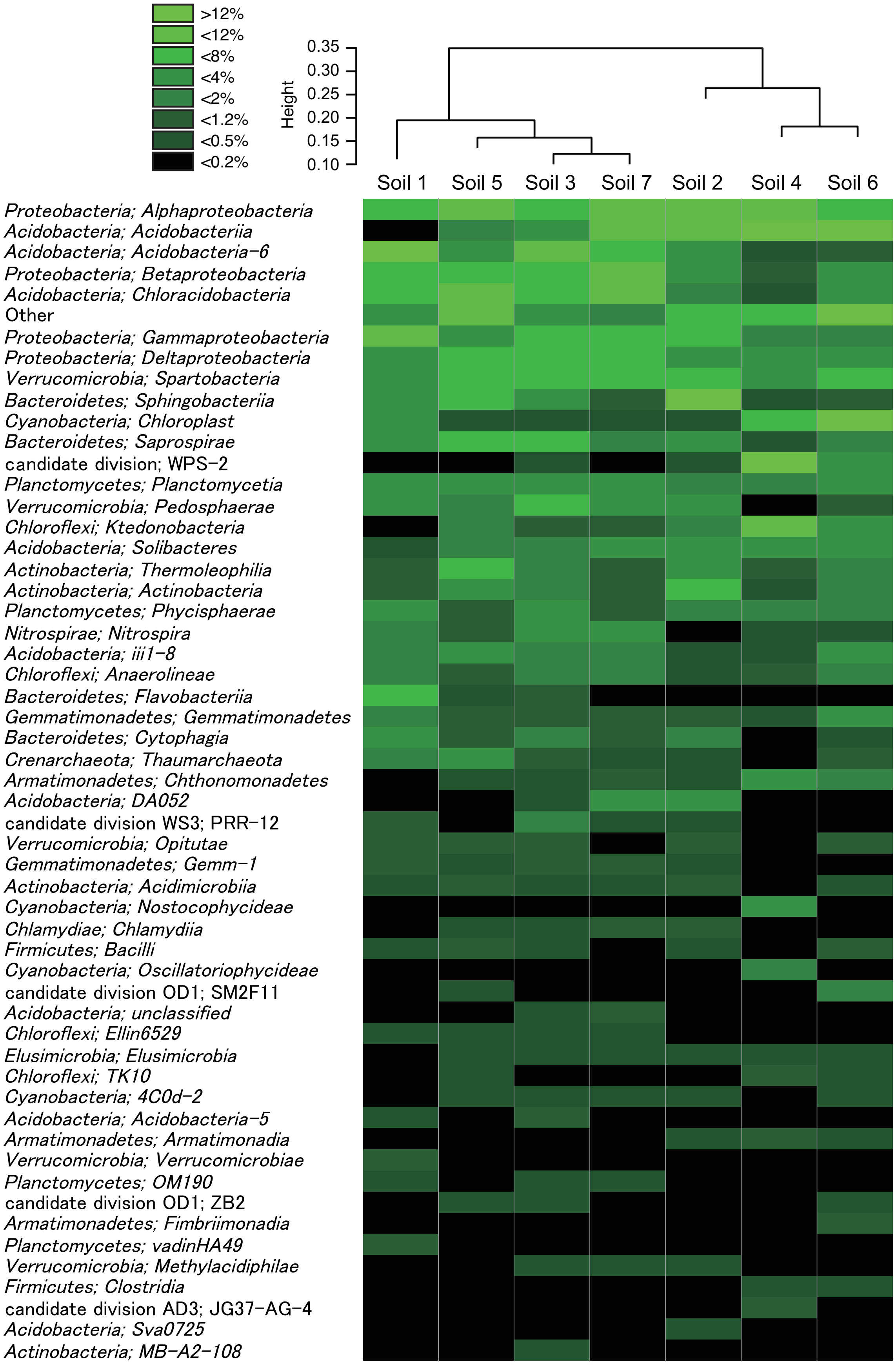

>12%
<12%
<8%
<4%
<2%
<1.2%
<0.5%
<0.2%
Height
0.35
0.30
0.25
0.20
0.15
0.10
Soil 1
Soil 5
Soil 3
Soil 7
Soil 2
Soil 4
Soil 6
Proteobacteria; Alphaproteobacteria
Acidobacteria; Acidobacteriia
Acidobacteria; Acidobacteria−6
Proteobacteria; Betaproteobacteria
Acidobacteria; Chloracidobacteria
Other
Proteobacteria; Gammaproteobacteria
Proteobacteria; Deltaproteobacteria
Verrucomicrobia; Spartobacteria
Bacteroidetes; Sphingobacteriia
Cyanobacteria; Chloroplast
Bacteroidetes; Saprospirae
candidate division; WPS−2
Planctomycetes; Planctomycetia
Verrucomicrobia; Pedosphaerae
Chloroflexi; Ktedonobacteria
Acidobacteria; Solibacteres
Actinobacteria; Thermoleophilia
Actinobacteria; Actinobacteria
Planctomycetes; Phycisphaerae
Nitrospirae; Nitrospira
Acidobacteria; iii1−8
Chloroflexi; Anaerolineae
Bacteroidetes; Flavobacteriia
Gemmatimonadetes; Gemmatimonadetes
Bacteroidetes; Cytophagia
Crenarchaeota; Thaumarchaeota
Armatimonadetes; Chthonomonadetes
Acidobacteria; DA052
candidate division WS3; PRR−12
Verrucomicrobia; Opitutae
Gemmatimonadetes; Gemm−1
Actinobacteria; Acidimicrobiia
Cyanobacteria; Nostocophycideae
Chlamydiae; Chlamydiia
Firmicutes; Bacilli
Cyanobacteria; Oscillatoriophycideae
candidate division OD1; SM2F11
Acidobacteria; unclassified
Chloroflexi; Ellin6529
Elusimicrobia; Elusimicrobia
Chloroflexi; TK10
Cyanobacteria; 4C0d−2
Acidobacteria; Acidobacteria−5
Armatimonadetes; Armatimonadia
Verrucomicrobia; Verrucomicrobiae
Planctomycetes; OM190
candidate division OD1; ZB2
Armatimonadetes; Fimbriimonadia
Planctomycetes; vadinHA49
Verrucomicrobia; Methylacidiphilae
Firmicutes; Clostridia
candidate division AD3; JG37−AG−4
Acidobacteria; Sva0725
Actinobacteria; MB−A2−108

Figure S3

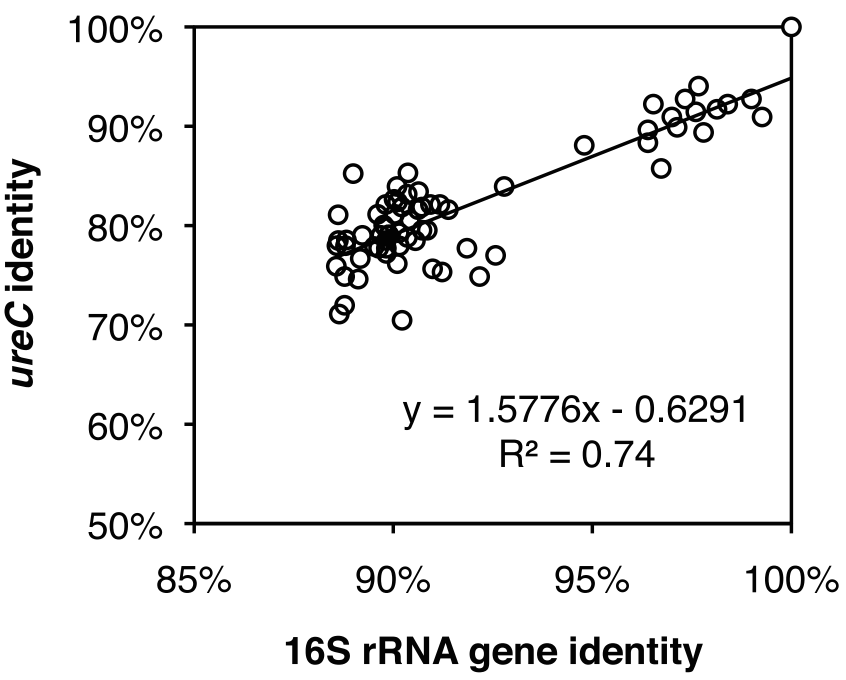
100%
90%
80%
70%
60%
50%
ureC identity
85%
90%
95%
100%
16S rRNA gene identity
y = 1.5776x - 0.6291
R² = 0.74

Figure S4

Supplementary Table Legends

Table S1. Description of the soil samples examined in the present study. C; total carbon, N; total nitrogen, NH_4^+; 0.5M sodium acetate-extractable ammonium, NO_2^-, NO_3^- and P; 0.002N H_2SO_4-extractable nitrite, nitrate, and phosphorus, K and Ca; 1M ammonium acetate-extractable potassium and calcium, and Fe; 1M KCl-extractable iron.

		pH	water contents	C ($g kg^{-1}$)	N ($g kg^{-1}$)	NH_4^+ ($mg kg^{-1}$)	NO_2^- ($mg kg^{-1}$)	NO_3^- ($mg kg^{-1}$)	P ($mg kg^{-1}$)	K ($mg kg^{-1}$)	Ca ($mg kg^{-1}$)	Fe ($mg kg^{-1}$)
Soil 1	agricultural soil (*Colocasia esculenta*)	7.4	33%	25	1.0	3.3	0.27	35	5.1	110	2555	<5
Soil 2	forest soil (*Japanese ceder*)	5.4	23%	26	1.3	4.3	<0.1	21	0.20	116	691	<5
Soil 3	glassland soil	6.4	25%	2.6	1.0	1.9	<0.1	28	0.78	124	661	<5
Soil 4	urban park soil	4.5	17%	1.6	0.4	1.5	<0.1	8	0.07	34.3	174	14
Soil 5	compost soil (kitchen garbage)	7.4	27%	34	1.6	0.9	<0.1	36	0.36	73.3	1116	21
Soil 6	freshwater sediment	5.1	29%	3.2	1.2	20	<0.1	18	0.19	47.5	277	28
Soil 7	forest soil (bamboo)	9.4	33%	40	1.6	2.1	<0.1	53	0.55	103	935	7.5

Table S2. Community richness, diversity, and evenness indices of the soils analysed in the present study. OTU: operational taxonomic unit. ±: standard deviation.

a) 16S rRNA gene

	read	OTU	Shannon	Simpson	Chao1	Good's coverage
Soil 1	13290	1837	9.1	0.990 ± 0.0003	4025 ± 147	79.6% ± 0.29%
Soil 2	11808	1896	9.4	0.993 ± 0.0003	4272 ± 128	78.6% ± 0.26%
Soil 3	14731	2263	9.9	0.996 ± 0.0001	5469 ± 120	73.2% ± 0.22%
Soil 4	15342	1143	8.1	0.987 ± 0.0003	2157 ± 136	88.5% ± 0.43%
Soil 5	12684	2073	9.8	0.996 ± 0.0002	4239 ± 164	78.1% ± 0.34%
Soil 6	10300	1591	8.8	0.989 ± 0.0004	3045 ± 147	84.0% ± 0.41%
Soil 7	6638	1894	9.5	0.993 ± 0.00002	4680 ± 18	76.9% ± 0.02%

b) *ure*C

	read	OTU	Shannon	Simpson	Chao1	Good's coverage
Soil 1	3537	363	6.5	0.971 ± 0.0011	512 ± 40	94.6% ± 0.25%
Soil 2	3500	1426	9.1	0.993 ± 0.0002	3212 ± 233	67.7% ± 0.76%
Soil 3	3757	1438	9.4	0.995 ± 0.0002	2739 ± 152	70.2% ± 0.57%
Soil 4	2511	1149	9.0	0.992 ± 0.00004	3260 ± 26	66.9% ± 0.12%
Soil 5	4299	1385	8.6	0.981 ± 0.0009	2472 ± 165	73.5% ± 0.71%
Soil 6	3333	731	7.8	0.984 ± 0.0004	1204 ± 85	85.7% ± 0.48%
Soil 7	6248	1685	8.8	0.985 ± 0.0007	2157 ± 183	76.0% ± 0.71%

Table S3. The closest relatives of the 34 most abundant operational taxonomic units (OTUs) of *ureC* (corresponding to the species level). A blastn search was carried out using the nucleic acid sequence of each species level OTU of *ureC* as a query sequence and the nr database (National Center for Biotechnology Information) as reference sequences. Nucleotide sequence accession numbers are indicated in parentheses.

ureC OTU	Closest relative	identity	*e*-value
2 (LC287204)	*Delftia tsuruhatensis* CM13 (CP017420.1)	81%	8.5×10^{-87}
283 (LC280142)	*Pseudomonas* sp. UW4 (CP003880.1)	95%	3.9×10^{-148}
505 (LC280352)	*Rhizobacter gummiphilus* NS21 (CP015118.1)	87%	7.5×10^{-113}
507 (LC280353)	*Caldilinea aerophila* (AP012337.1)	75%	6.3×10^{-57}
1050 (LC280858)	*Caldilinea aerophila* (AP012337.1)	77%	1.1×10^{-53}
1144 (LC280942)	*Nitrospira japonica* NJ11 (LT828648.1)	88%	8.0×10^{-119}
1290 (LC281081)	*Afipia* sp. GAS231 (LT629703.1)	93%	7.0×10^{-145}
1384 (LC281171)	*Immundisolibacter cernigliae* TR32 (CP014671.1)	81%	6.2×10^{-76}
1435 (LC281221)	*Thermobacillus composti* KWC4 (CP003255.1)	76%	7.5×10^{-56}
1885 (LC287326)	*Agrobacterium* sp. RAC06 (CP016499.1)	89%	7.0×10^{-126}
2335 (LC282065)	*Cupriavidus metallidurans* CH34 (CP000352.1)	84%	5.4×10^{-102}
2444 (LC282169)	*Burkholderia* sp. BDU6 (CP013386.1)	77%	2.0×10^{-69}
2460 (LC282184)	*Pseudomonas* sp. MT-1 (AP014655.1)	81%	2.5×10^{-87}
2477 (LC282201)	*Methyloversatilis* sp. RAC08 (CP016448.1)	79%	9.1×10^{-74}
2606 (LC282317)	*Thauera* sp. MZ1T (CP001281.2)	83%	9.7×10^{-99}
2826 (LC282520)	*Bradyrhizobium erythrophlei* GAS138 (LT670817.1)	83%	4.1×10^{-97}
2846 (LC282540)	*Herbaspirillum frisingense* AA6 (CP018845.1)	84%	3.4×10^{-98}
3157 (LC287405)	Uncultured t*haumarchaeote* clone B1-C-21 (KM525691.1)	80%	1.5×10^{-83}
3295 (LC282957)	*Candidatus* Nitrososphaera evergladensis SR1 (CP007174.1)	78%	9.1×10^{-74}
3313 (LC282974)	*Ralstonia solanacearum* IBSBF1503 (CP012943.1)	81%	2.0×10^{-69}
3907 (LC283528)	*Pseudomonas brassicacearum* BS3663	95%	3.9×10^{-148}

ureC OTU	Closest relative	identity	*e*-value
	(LT629713.1)		
3974 (LC283587)	*Bradyrhizobium* sp. S23321 (AP012279.1)	93%	5.7×10^{-146}
4155 (LC283757)	Uncultured archaeon clone JD HF 10 (KM396444.1)	83%	1.5×10^{-83}
4596 (LC284169)	*Burkholderia multivorans* MSMB1640WGS (CP013467.1)	78%	8.0×10^{-62}
4606 (LC287507)	Uncultured archaeon clone LZ HF 6 (KM396450.1)	80%	1.5×10^{-83}
4747 (LC284312)	*Burkholderia oklahomensis* strain 1974002358 (CP013358.1)	85%	2.5×10^{-106}
4796 (LC284354)	*Cupriavidus basilensis* 4G11 (CP010536.1)	84%	1.9×10^{-101}
4838 (LC284394)	*Bradyrhizobium canariense* GAS369 (LT629750.1)	87%	9.7×10^{-118}
5062 (LC284606)	*Bradyrhizobium erythrophlei* GAS242 (LT670818.1)	90%	1.4×10^{-128}
6797 (LC286227)	*Burkholderia glumae* (CP009435.1)	82%	4.7×10^{-90}
6801 (LC286230)	*Cylindrospermum stagnale* PCC7417 (CP003642.1)	89%	4.4×10^{-103}
7222 (LC286630)	*Bradyrhizobium* sp. BTAi1 (CP000494.1)	87%	1.4×10^{-115}
7277 (LC286681)	*Nitrospira moscoviensis* NSP M-1 (CP011801.1)	80%	1.0×10^{-85}
7572 (LC286956)	*Bradyrhizobium diazoefficiens* (AP014685.1)	92%	5.4×10^{-140}

Supplementary text

The following supplementary text includes a detailed protocol of the experiments conducted in the present study.

1. DNA extraction and quantitative PCR

Genomic DNA was extracted using the PowerSoil DNA Isolation kit (MO BIO Laboratories). DNA concentrations were determined using the Qubit dsDNA BR assay kit and a Qubit 3.0 fluorospectrometer (Thermo Fisher Scientific). The qPCR assay was performed using oligonucleotide primers 515F (5′- GTGCCAGCMGCCGCGGTAA-3′) and 806R (5′-GGACTACHVGGGTWTCTAAT-3′) for the 16S rRNA gene (1) and L2F_V1 (5′-CGGCAAGGCCGGCAACCC-3′) and 733R (5′-GTBGHDCCCCARTCYTCRT-3′) for *ureC* (9). Sequences of primers L2F_V1 and 733R were taken from another study with minor modifications. Degenerate bases at positions +3, +6, +9, +12, +15, and +18 relative to the 5′ terminus of the original L2F primer were modified to decrease sequence complexity, i.e. H to C at position +3, Y to C at +6, R to G at +9, N to C at +12, N to C at +15, and Y to C at +18. The PCR mixture had a volume of 20 µl and contained 2 ng of an extracted DNA sample or 2 µl of standard DNA, oligonucleotide primers (0.3 µM each), and 1×SSoFast EvaGreen Supermix (Bio-Rad). The cycling conditions were as follows: 98°C for 2 min; 40 cycles at 98°C for 5 s and 50°C for 10 s; and finally, 65°C to 95°C with 0.5°C increments for melting curve analysis. The assay was conducted in triplicate on a MiniOpticon thermal cycler (Bio-Rad), and specific amplification of the 16S rRNA gene and of *ureC* was ascertained by agarose gel electrophoresis of the amplicons. Genomic DNA of *Pseudomonas aeruginosa* PAO1 (JCM14847) with 4 and 1 copies of the 16S rRNA gene and *ureC*, respectively, was used as a standard for quantification. The cells were

cultured as recommended by the supplier, genomic DNA was extracted, and DNA concentration was determined as mentioned above. The genomic DNA was serially diluted with distilled water to concentrations of 10^5 to 10^0 copies·µl^{-1}.

2. Amplicon sequencing of the 16S rRNA gene and ur*e*C

PCR amplification of the 16S rRNA gene and of *ureC* was carried out using the above-mentioned oligonucleotide primers containing Illumina tag sequences at the 5′ end of the forward and reverse primers (5′- TCGTCGGCAGCGTCAGATGTGTATAAGAGACAG-3′ and 5′-GTCTCGTGGGCTCGGAGATGTGTATAAGAGACAG-3′, respectively). The PCR mixture had a volume of 30 µl and contained 30 ng of an extracted DNA sample, the oligonucleotide primers (0.5 µM each), dNTPs (200 µM), 1× PCR buffer, and ExTaq polymerase (0.025 U·µl^{-1}). The cycling conditions were as follows: 35 and 40 cycles for the 16S rRNA gene and *ureC*, respectively, at 98°C for 10 s, followed by 55°C for 30 s, then 72°C for 30 s; and finally, 72°C for 10 min. PCR products were purified using the FastGene Gel/PCR Extraction Kit (Nippon Genetics). The purified PCR products were tagged with a sample-unique index and Illumina adapter sequences at their 5′ end (Nextera XT Index Kit v2, Illumina) by PCR. The PCR reaction mixture (20 µl) contained 1× KAPA HiFi HS ReadyMix (Kapa Biosystems), 1 µl of each forward and reverse primer (10 µM), and 2 µl of the recovered PCR products. The PCR was run under the following cycling conditions: 95°C for 3 min, 10 cycles of 95°C for 20 s, 65°C for 15 s, and 72°C for 1 min; and finally, 72°C for 5 min. After agarose gel electrophoresis, the PCR products were excised from the gel and purified using an Agencourt AMPure XP Kit (Beckman Coulter). The tagged amplicons were pooled and sequenced on the Illumina MiSeq platform in a 250-bp paired-end sequencing reaction with the v2 reagent kit (Illumina).

3. Bioinformatics

The generated *ureC* and 16S rRNA gene sequence reads were processed for removal of adapter sequences using cutadapt and for quality trimming using Trimmomatic v0.33 (1) as previously described (10). The reads that contained <50 bp or were associated with an average Phred-like quality score <30 were removed. Paired-end sequence reads were assembled in the paired-end assembler for the Illumina sequence software package (PANDAseq) (12). The obtained *ureC* reads were subjected to a blastn search (threshold *e*-value; 10^{-10}) against the known 60,733 *ureC* sequences downloaded from fungene database (8) and the database of Integrated Microbial Genomes & Microbiome Samples (IMG/MER) (11) to remove non-*ureC* sequences. As for the 16S rRNA gene, the assembled sequence reads with ≥97% sequence identity were grouped into an OTU by UCLUST (6). Phylogenetic affiliations of the OTUs were identified using a blastn search against reference sequences in the Greengenes database version 13_5 (4) and in the nr database (National Center for Biotechnology Information). As for *ureC*, sequence reads with ≥91% sequence identity were grouped into an OTU, and the phylogenetic affiliation was examined using a blastn search in the nr database. Putative chimeric sequences were removed using UCHIME (7). Alpha diversity indices (observed species, Chao1, Good's coverage, and Simpson's index) were calculated in QIIME (2). Chao1 was computed at a sampling depth of 5,500 reads and 2,500 reads for the 16S rRNA gene and *ureC* gene, respectively. A phylogenetic tree was constructed using the nucleic acid sequences of *ureC* by the maximum likelihood method with the Jones-Taylor-Thornton model in the MEGA 6.06 software (14). Cluster analysis was carried out to examine similarities of community composition among the soils using the STAMP software (13).

4. Evaluation of ureC primers for PCR amplification of the known ureC sequences

Coverage of the previously designed *ure*C primers was examined by aligning known *ure*C sequences and the *ure*C primer sequences, and by counting the primer–template mismatches. A total of 17,312 *ure*C sequences were downloaded from the database of IMG/MER (11). We found that the *ure*C sequences derived from some bacterial species were tremendously abundant because many genome sequences have been determined and deposited, e.g. 3,174 genomes were deposited in the database entry for the species *Escherichia coli* (accessed on 16th May 2017). On the other hand, for the majority of bacterial species, only a limited number of genome sequences was available. To evaluate the coverage of *ure*C primers for phylogenetically distinct *ure*C sequences uniformly, the *ure*C sequences derived from the genome sequences affiliated with the same bacterial species were grouped, and a representative *ure*C sequence was subjected to sequence alignment. Alignment of 2,653 *ure*C sequences was performed using the MUSCLE software under default conditions (16 iterations) (5). Regions in which primers L2F_V1, 733R, ureC_F, and ureC_R hybridised were manually examined, and primer–template mismatches were counted.

References

1. Bolger, A. M., M. Lohse and B. Usadel. 2014. Trimmomatic: a flexible trimmer for Illumina sequence data. Bioinformatics. 30:2214-2120.
2. Caporaso, J. G., J. Kuczynski, J. Stombaugh et al. 2010. QIIME allows analysis of high-throughput community sequencing data. Nature methods. 7:335-336.
3. Caporaso, J.G., C.L. Lauber, W.A. Walters, D. Berg-Lyons, C.A. Lozupone, P.J. Turnbaugh, N. Fierer and R. Knight. 2011. Global patterns of 16S rRNA diversity at a depth of millions

of sequences per sample. Proc. Natl. Acad. Sci. U. S. A. 108:4516-4522.

4. DeSantis, T. Z., P. Hugenholtz, N. Larsen et al. 2006. Greengenes, a chimera-checked 16S rRNA gene database and workbench compatible with ARB. Appl. Environ. Microbiol. 72:5069–5072.

5. Edgar, R. C. 2004. MUSCLE: Multiple sequence alignment with high accuracy and high throughput. Nucleic Acids Res. 32:1792-1797.

6. Edgar, R. C. 2010. Search and clustering orders of magnitude faster than BLAST. Bioinformatics. 26:2460–2461.

7. Edgar, R. C., B.J. Haas, J.C. Clemente, C. Quince and R. Knight. 2011. UCHIME improves sensitivity and speed of chimera detection. Bioinformatics. 27:2194–2200.

8. Fish, J.A., Chai, B., Wang, Q. *et al.* 2013. FunGene: the functional gene pipeline and repository, Front. Microbiol. 4:291.

9. Fujita, Y., J.L. Taylor, L.M. Wendt, D.W. Reed and R.W. Smith. 2010. Evaluating the potential of native ureolytic microbes to remediate a ^{90}Sr contaminated environment. Environ. Sci. Technol. 44:7652-7658.

10. Hirakata, Y., M. Oshiki, K. Kuroda, M. Hatamoto, K. Kubota, T. Yamaguchi, H. Harada and N. Araki. 2016. Effects of predation by protists on prokaryotic community function, structure, and diversity in anaerobic granular sludge. Microbes Environ. 31:279-287.

11. Markowitz, V.M., I.M.A. Chen, K. Palaniappan et al.. 2010. The integrated microbial genomes system: an expanding comparative analysis resource. Nucleic Acids Res. 38:D382-D390.

12. Masella, A.P., A.K. Bartram, J.M. Truskowski, D.G. Brown and J.D. Neufeld. 2012.

PANDAseq: paired-end assembler for illumina sequences. BMC Bioinformatics 13:1-7.

13. Parks, D. H., G.W. Tyson, P. Hugenholtz and R.G. Beiko. 2014. STAMP: statistical analysis of taxonomic and functional profiles. Bioinformatics. 30:3123-3124.

14. Tamura, K., G. Stecher, D. Peterson, A. Filipski and S. Kumar. 2013. MEGA6: Molecular evolutionary genetics analysis version 6.0. Mol. Biol. Evol. 30:2725-2729.
